# Immunomodulatory Effect of Colistin and its Protective Role in Rats with Methicillin-Resistant *Staphylococcus aureus-*induced Pneumonia

**DOI:** 10.3389/fphar.2020.602054

**Published:** 2021-01-20

**Authors:** Hui Niu, Tianli Yang, Jin Wang, Rui Wang, Yun Cai

**Affiliations:** Department of Pharmacy, Center of Medicine Clinical Research, Medical Supplies Center of PLA General Hospital, Beijing, China

**Keywords:** colistin (MeSH), immunomodulatory, MRSA, pneumonia, cytokines

## Abstract

**Objectives:** Colistin is the last resort of antimicrobials against multi-drug resistant Gram-negative pathogens. Previous studies in *Caenorhabditis elegans* and macrophages of rats have suggested that colistin possesses the immunomodulatory properties by acting p38/MAPK pathway. Here, we aimed to confirm the immunomodulatory role of colistin in animal models.

**Methods:** Rat model of Methicillin-resistant *Staphylococcus aureus* (MRSA)-induced pneumonia was established. Plasma concentrations of proinflammatory cytokines, quantitative bacteriology, histology and immunohistochemistry of lungs were assessed to compare the immunomodulatory properties of colistin pre-administration.

**Results:** The numbers of white blood cells and granulocytes were significantly increased in the 9 mg/kg colistin pre-administration group at 72 h after infection. Levels of TNF-α, IL-6 and IL-1β in plasma after colistin pre-administration were lower compared with the infected group without treatment. Colistin pre-treatment resulted in lower bacterial counts, a dramatic decrease of cytokines and improved histopathological injury in infected lung tissues compared with the untreated animals. However, p38/MAPK inhibitor SB203580 did not fully block the above-mentioned effects caused by colistin.

**Conclusion:** Pre-administration of colistin could attenuate an excessive inflammatory reaction and protect the lungs from MRSA-associated damages. However, these effects could not be reversed by blocking the p38/MAPK pathway alone. Collectively, the mechanism underlying the immunoregulatory effects of colistin in mammals needs to be further explored.

## Introduction

Multidrug-resistant (MDR) and extensively drug-resistant (XDR) Gram-negative bacteria have become an increasing challenge in antibiotic resistance, posing a global risk to public health (Tacconelli et al., 2018). Clinicians and scientists have to use old antibiotics and reassess their clinical use in order to deal with above-mentioned resistant organisms due to the lack of new antimicrobials and therapeutics. Among these old drugs, colistin is undoubtedly one of the most outstanding ones. The latest International Network for Optimal Resistance Monitoring (INFORM) surveillance shows that susceptibility to colistin and tigecycline (77.0 and 78.1%, respectively) is similar to that of ceftazidime-avibactam (73.0%) among meropenem-non-susceptible Enterobacteriaceae. Moreover, colistin has been shown to be the most active antimicrobial against Metallo-β-lactamase (MBL)-positive isolates, surpassing tigecycline and ceftazidime-avibactam which is inactive against MBL-positive isolates (Spiliopoulou et al., 2020). Another global surveillance program has compared the antimicrobial activity of ceftolozane-tazobactam with other antibiotics against *Pseudomonas aeruginosa* isolates from 104 hospitals on four continents. They have found that ceftolozane-tazobactam is more active than all comparators, except for colistin. The susceptibility to ceftolozane-tazobactam and colistin in all *P. aeruginosa* strains and MDR stains is 93.5 vs. 99.8% and 69.2 vs. 99.3%, respectively (Shortridge et al., 2019).

Colistin is a multicomponent polypeptide antibiotic, which is isolated in 1950 from the *Bacillus polymyxa* var. *Colistinus* Koyama (Ito and Koyama 1972). Due to neurotoxicity and nephrotoxicity, the application of colistin is restricted during 1970s. In the past decade, colistin has re-emerged in clinical practice as last resort to treat infections caused by MDR and XDR Gram-negative strains, including non-fermentative bacteria (*P. aeruginosa*, *Acinetobacter baumannii*, and *Stenotrophomonas maltophilia*) and Enterobacteriaceae (Li et al., 2006). Currently, we have entered the third era of anti-infective strategy, which intends to favor the interplay between the active molecules and immune system (Labro, 2012). More and more studies have shown that antimicrobial agents have regulatory effects on immune system, which can either enhance the defense ability against pathogens or inhibit the inflammatory response induced by infection (Uzun et al., 2014; Gibson et al., 2017). In addition to in-depth reassessment of nephrotoxicity and neurotoxicity, the research on colistin has also been expanded to explore its immunomodulatory effect. Previous study has confirmed that colistin has potential immune-stimulating effects on *Caenorhabditis elegans*. Colistin protects the host against infections by a conserved p38/PMK-1 pathway in the intestine of nematode, which is independent of its antimicrobial activity (Cai et al., 2014). Further research demonstrates that colistin increases the secretion of cytokines and phagocytotic ability of macrophages from rats. Furthermore, p38/MAPK pathway is also involved in such colistin-induced immunomodulatory effect (Wang et al., 2019). However, the immunoregulatory effect of colistin in living mammals has not been reported. In the present study, we aimed to investigate the role of colistin in the immune system of healthy and pneumonic rats.

## Materials and Methods

### Reagents

Colistin sulfate salt (Catalog no. C-4461), hematoxylin and eosin were purchased from Sigma-Aldrich (United States). p38/MAPK inhibitor SB203580 (Catalog no. HY-10256) was obtained from MedChemExpress (United States). ELISA kits for rat TNF-α, INF-γ, IL-1β and IL-6 (Catalog no. RTA00, RIF00, RLB00, R6000B) were supplied by R&D Systems (Catalog no. United States). Antibodies against IL-6, IL-1β and TNF-α (Catalog no. ab208113, ab239517, ab109322) were purchased from Abcam (United States). 3,3-Daminobenzidine (DAB) staining kit (Catalog no. SK-4100) was purchased from Vector Laboratories (United States).

### Animals

Male Sprague Dawley (SD) rats weighing 240-260 g (7–8 weeks) were housed in independent ventilation cages and permitted to adapt to their environment for 3 days prior to procedures. All animals received free access to food and water throughout the study. All procedures were approved by Animal Care and Use Committee (IACUC) of PLA General Hospital (No. 2017-X3-51) and all efforts were made to minimize the suffering of animals.

### Determination of Colistin Dosage

To characterize the immunomodulatory effects of colistin on health rats, 48 rats were used in preliminary studies to determine the appropriate colistin dosages and observation timepoints. Rats were randomly and evenly divided into eight groups as follows: single-dose control group (intraperitoneally injected with saline), single-dose colistin groups (intraperitoneally injected with 3, 6 or 9 mg/kg of colistin), twice-dose control group (intraperitoneally injected with saline, q12h), and twice-dose colistin groups (intraperitoneally injected with 1.5, 3 or 4.5 mg/kg of colistin, q12h). Rats were euthanized, and the blood samples were collected from heart into EDTA-2K anticoagulant vacuum tubes at 6 h post-administration for single-dose groups or 24 h after first dosage for twice-dose groups. Total white blood cells (WBCs) and differential count (including lymphocytes, granulocytes and monocytes) was detected from 2 ml whole blood using an automated hematology analyzer (Mindray, China) within 2 h after collection. The plasma was centrifuged (1,000 × g, 20 min, 4°C) from 5 ml whole blood within 30 min and rapidly stored at −80°C waiting for cytokines detection with ELISA kits.

### Bacterial Preparation for Inoculation

The Methicillin-resistant *Staphylococcus aureus* (MRSA) clinical isolate B3180 was obtained from PLA General Hospital. To prepare an animal inoculum, a frozen stock of MRSA B3180 was subcultured onto Mueller-Hinton Agar (MHA) and incubated overnight in Mueller-Hinton Broth (MHB) at 37°C. The overnight culture was diluted with fresh MHB to reach a final concentration of 3.0 McFarland standard (∼9 × 10^8^ CFU/ml).

### Rat Model of Methicillin-Resistant *Staphylococcus aureus*-Induced Pneumonia

Rat pneumonia model was established as described (Huang et al., 2018) to evaluate the immunomodulatory effect of colistin. MRSA was used because it is intrinsically resistant to colistin. Briefly, after intraperitoneally anesthetized with 1% pentobarbital sodium (XiangBo Biotechnology Co., Ltd. Guangdong, China) solution (50 mg/kg), the rat was placed on an angle-adjustable workstand and suspended by its incisors. The opening and closing of glottis could be observed by oto speculum. Subsequently, a single-use orotracheal intubation catheter (Hallowell EMC PN 000A3747 Mouse Intubation Pack, Pittsfield, United States) was inserted into the trachea through glottis. Then 300 µL MRSA suspension at 3.0 McFarland standard was slowly injected into the trachea. The total inoculation concentration was 2.7 × 10^8^ CFU/rat. Finally, the rat was vertically held on the workstand for 20 sec before being put back into the cage to recover from anesthesia.

### Experimental Groups of Rat Pneumonia Model

SD rats were randomly divided into five groups as follows: 1) negative control group (n = 6): pre-conditioned with vehicle solution and then inoculated with sterile broth at 0 h. 2) positive control group (n = 9): pre-conditioned with vehicle solution and then inoculated with MRSA at 2.7 × 10^8^ CFU/rat at 0 h; 3) colistin group (n = 9): pre-conditioned with colistin and then inoculated with MRSA; 4) SB203580 group (n = 9): pre-administrated administered with p38-inhibitor, SB203580, prior to pre-conditioning with vehicle solution and then inoculated with MRSA at 0 h; and 5) SB203580 + colistin group (n = 9): pre-administrated administered with SB203580 prior to pre-conditioning with colistin and then inoculated with MRSA at 0 h. The process of blood sampling, WBCs and cytokines detection was performed as previously described.

### Bacterial Quantification, Histology, Immunohistochemistry of Lungs

The executed rats were placed on the clean bench to dissect the lungs. Bacterial quantification was performed in left lung tissues, and pathological scoring and immunohistochemistry were performed in right lung tissues.

Left lung weighing 15-30 g was homogenized in 2 ml of saline. Subsequently, 200 μL lung homogenate and its serial dilutions were quantified by spreading a certain amount of dilutions on MHA and enumerating the colonies. Bacterial counts were calculated and expressed as the number of CFU per gram of tissue.

Right lung was fixed in 10% formalin solutions for 24 h, embedded in paraffin and cut into 4-µm sections. For histopathological observation, the slides were stained with hematoxylin and eosin (H and E) and examined under light microscope. Each slide was evaluated by two investigators who were blinded to the experimental design. Lung injury was evaluated based on a modified scoring system, including four categories consisting of edema, hemorrhage, leukocyte infiltration and alveolar septal thickening (Yamanel et al., 2011). Each category was scored from 0 to 4. Total lung injury score was calculated by summing the individual scores for each category. The scores for each histological parameter were summed up to a maximum score of 16. The scoring results were expressed as total lung injury score. For IHC evaluation, the slides were deparaffinized and incubated in 3% hydrogen peroxide/methanol at room temperature for 10 min to inhibit endogenous peroxidase activity. Then tissue sections were incubated with primary antibodies (rabbit anti-rat polyclonal IL-6, IL-1β and TNF-α at a dilution of 1:8,000 respectively) at 4°C overnight. The slides were repeatedly washed with PBS, followed by incubation with an goat anti-rabbit secondary antibody conjugated with horseradish peroxidase at room temperature for 1 h. The secondary antibody was visualized using the DAB staining kit according to the manufacturer’s instructions. The slides were counterstained with hematoxylin to determine the presence of nuclei. Finally, dark brown cells were considered to be positive staining. Photomicrographs were taken with Nikon Eclipse Ci-S (Nikon Instruments Inc., Melville, NY). The relative density of immunostaining (density/area) was determined using Image-Pro Plus 6.0 Software.

### Statistical Analysis

Results were expressed as mean ± standard error of mean (SEM). Analysis of numerical data was performed by one-way analysis of variance (ANOVA) and Student’s t test. *p* value <0.05 was considered as statistically significant.

## Results

### Colistin Increases White Blood Cells Counts and Cytokine Levels in Healthy Rats

**FIGURE 1:**
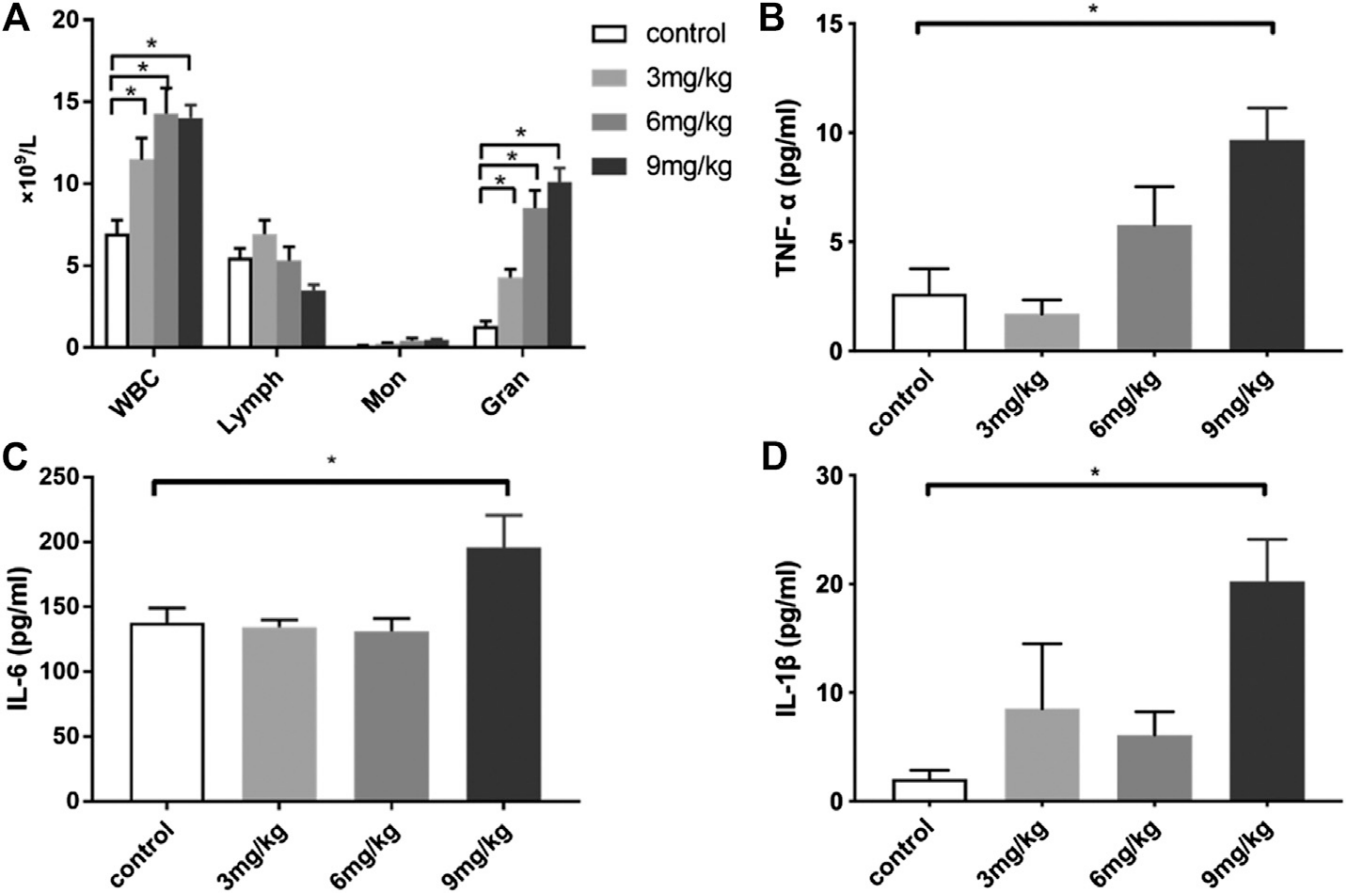
The changes of WBC, WBC differential count and cytokines levels at 6 h after single dose of colistin in healthy rats. **(A)** Total WBC, lymphocyte, monocyte, neutrophil count; **(B)** TNF-α; **(C)** IL-6; **(D)** IL-1β. Six animals in each group. Data are presented as mean ± SEM, *p < 0.05 compared to control group.

To establish the optimal dosage regimen and observation timepoints for immunomodulatory effect of colistin, healthy rats were treated with single dosage at 3, 6 or 9 mg/kg or twice dosage at 1.5, 3 or 4.5 mg/kg q12h. At 24 h after the first dosage, no significant difference was found in WBC counts, WBC differential counts and the level of inflammatory cytokine TNF-α in healthy rats, which received colistin twice daily (data not shown). By contrast, the inflammatory response of rats in single-dosage colistin group was obviously changed at 6 h. Total WBC counts and granulocyte counts of 3, 6 or 9 mg/kg single-dosage colistin groups were significantly increased compared with the control group (*p* < 0.05). The levels of TNF-α, IL-6 and IL-1β in plasma of the 9 mg/kg single-dosage colistin group were significantly higher compared with the control group (*p* < 0.05), while the cytokine levels of 3 and 6 mg/kg single-dosage colistin groups were not significantly increased (Figure 1).

### Pre-administration of Colistin in Methicillin-Resistant *Staphylococcus aureus*-Induced Pneumonic Rats

Six rats in the negative control group were inoculated with broth. A total of 36 rats in the other four groups were intratracheally inoculated with 2.7 × 10^8^ CFU/rat MRSA. According to the results from healthy rats, obvious immunomodulatory effect could be observed at 6 h after administration of 9 mg/kg colistin. Therefore, colistin or vehicle solution was intraperitoneally injected at 6 h prior to inoculation. Moreover, p38/MAPK inhibitor SB203580 was intraperitoneally injected at 7 h before inoculation. All animals were monitored until 72 h after inoculation. There were no surgically-related deaths within 2 h after inoculation. One rat died before 72 h in the positive control group, colistin group and SB203580 + colistin group, respectively. No death occurred in the negative control group or SB203580 group. No lung tissues and blood samples were taken from the dead rats. Detailed treatment regimen was listed in Table 1.

**TABLE 1:** Treatment regimen of colistin in MRSA-induced pneumonic rats.

Groups	Total rats	−7 h i.p.	−6 h i.p.	0 h intratracheal inoculation	Alive rats 72 h after inoculation
Negative control	6	—	Water for injection	Broth	6
Positive control	9	—	Water for injection	MRSA	8
Colistin	9	—	9 mg/kg colistin	MRSA	8
SB203580	9	5 mg/kg SB203580	Water for injection	MRSA	9
SB203580 + colistin	9	5 mg/kg SB203580	9 mg/kg colistin	MRSA	8

i.p. = intraperitoneal injection.

### Pre-administration of Colistin Increases the White Blood Cells Counts of Pneumonic Rats

**FIGURE 2:**
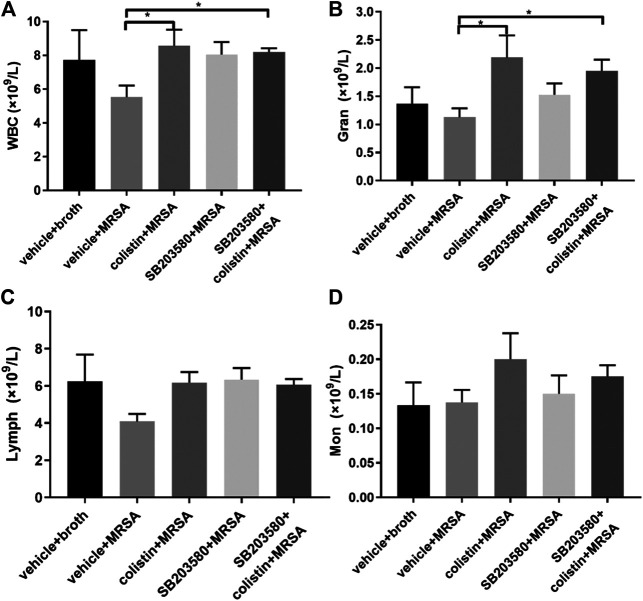
The changes of WBC and cytokines levels at 72 h after inoculation with MRSA in rat pneumonia model. **(A)** WBC; **(B)** neutrophil count; **(C)** lymphocyte; **(D)** monocyte. Negative control group: vehicle + broth (n = 6); positive control group: vehicle + MRSA (n = 8); colistin group: 9 mg/kg colistin + MRSA (n = 8); SB203580 group: 5 mg/kg SB203580 + MRSA (n = 9); SB203580 + colistin group: 5 mg/kg SB203580 + 9 mg/kg colistin + MRSA (n = 8). Data are presented as mean ± SEM, *p < 0.05 compared to positive control group.

At 72 h after intratracheal inoculation of MRSA, the total WBC counts in the colistin group and SB203580 + colistin group were significantly higher compared with the positive control group, while no significant difference was found between other groups (Figure 2A). The change of total WBC differential counts could be mainly attributed to the granulocytes, which showed the similar results with WBC counts (Figure 2B). No difference was found in lymphocytes and monocytes (Figures 2C,D).

### Pre-administration of Colistin Reduces the Methicillin-Resistant *Staphylococcus aureus* Growth in Lung Tissue of Pneumonic Rats

**FIGURE 3:**
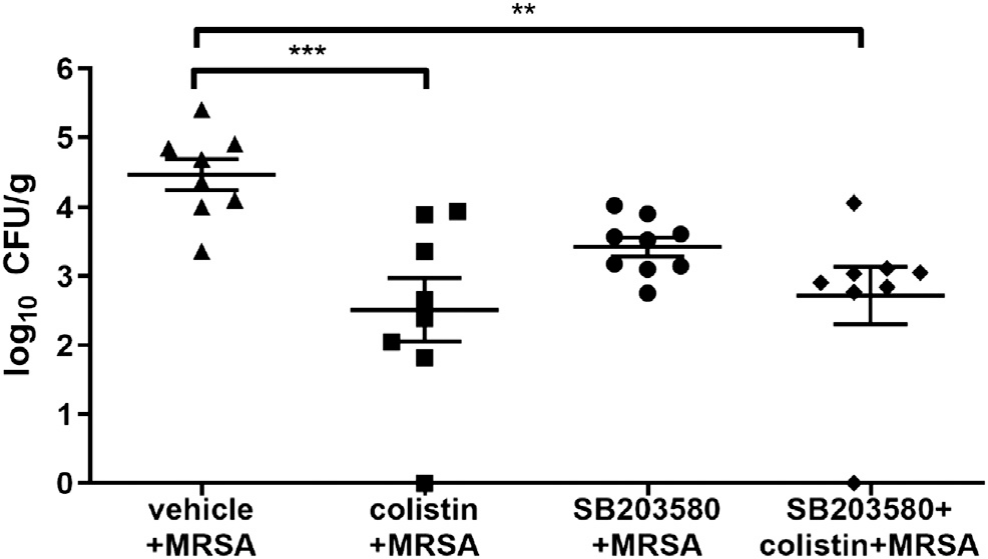
Quantification of bacteria in lung tissue at 72 h after inoculation with MRSA in rat pneumonia model. MRSA was intratracheally inoculated into the lungs at 2.7 × 108 CFU/rat. Each data point represents bacteria counts from a single rat. Horizontal lines represent the mean of the results from the same treatment set. Because no colony was found in vehicle + broth group, the results were not presented in this figure. **p < 0.01, ***p < 0.001 compared to vehicle + MRSA group.

To test whether pre-administration of colistin could affect the host defense, we examined the bacterial colony counts from lung tissues at 72 h after MRSA infection, which is intrinsically resistant to colistin. No colonies of MRSA were found from the lungs of rats in the negative control group. MRSA growth in lung tissues (CFU/g) was significantly lower in the colistin and SB203580 + colistin groups compared with the positive control group, while the bacterial growth was not different between these two groups. Moreover, the SB203580 group exhibited the similar level of colony counts compared with the positive group (Figure 3).

### Pre-administration of Colistin Affects the Cytokine Levels in Plasma of Infected Rats

**FIGURE 4:**
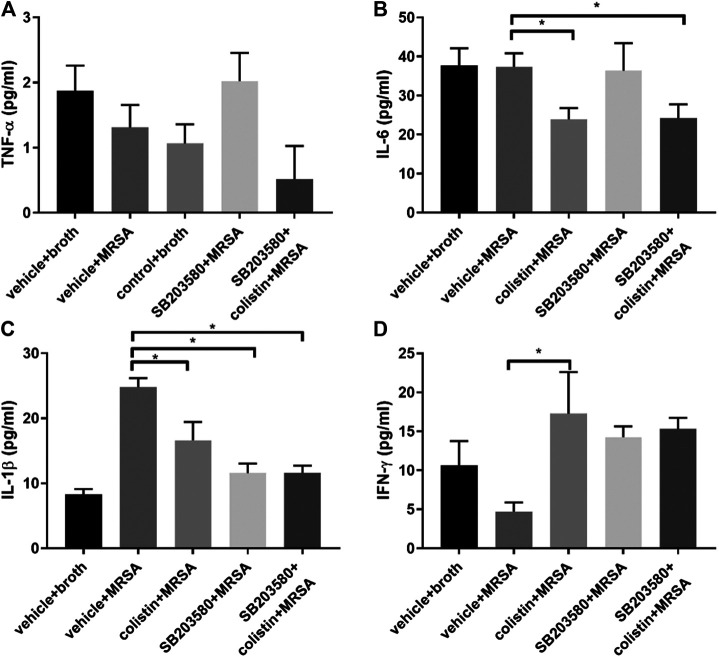
The changes of cytokines in blood at 72 h after inoculation with MRSA in rat pneumonia model. **(A)** TNF-α; **(B)** IL-6; **(C)** IL-1β; **(D)** IFN-γ. Negative control group: vehicle + broth (n = 6); positive control group: vehicle + MRSA (n = 8); colistin group: 9 mg/kg colistin + MRSA (n = 8); SB203580 group: 5 mg/kg SB203580 + MRSA (n = 9); SB203580 + colistin group: 5 mg/kg SB203580 + 9 mg/kg colistin + MRSA (n = 8). Data are presented as mean ± SEM, *p < 0.05 compared to positive control group.

The changes of cytokines at 72 h in MRSA-induced pneumonic rats demonstrated a different pattern compared with the healthy rats at 6 h. There was no significant difference in TNF-α among all five groups (Figure 4A). After 72 h of infection, the IL-6 level remained stable between the positive and negative control groups and was similar between the colistin and SB203580 + colistin groups, but was significantly lower compared with the positive control group (Figure 4B). The level of IL-1β in the positive control group was almost tripled compared with the negative control group and also significantly higher than that in the colistin, SB203580 and SB203580 + colistin groups (Figure 4C).

### Pre-administration of Colistin Prevents Lung Injury and Suppresses Methicillin-Resistant *Staphylococcus aureus*-Induced Lung Inflammation

**FIGURE 5:**
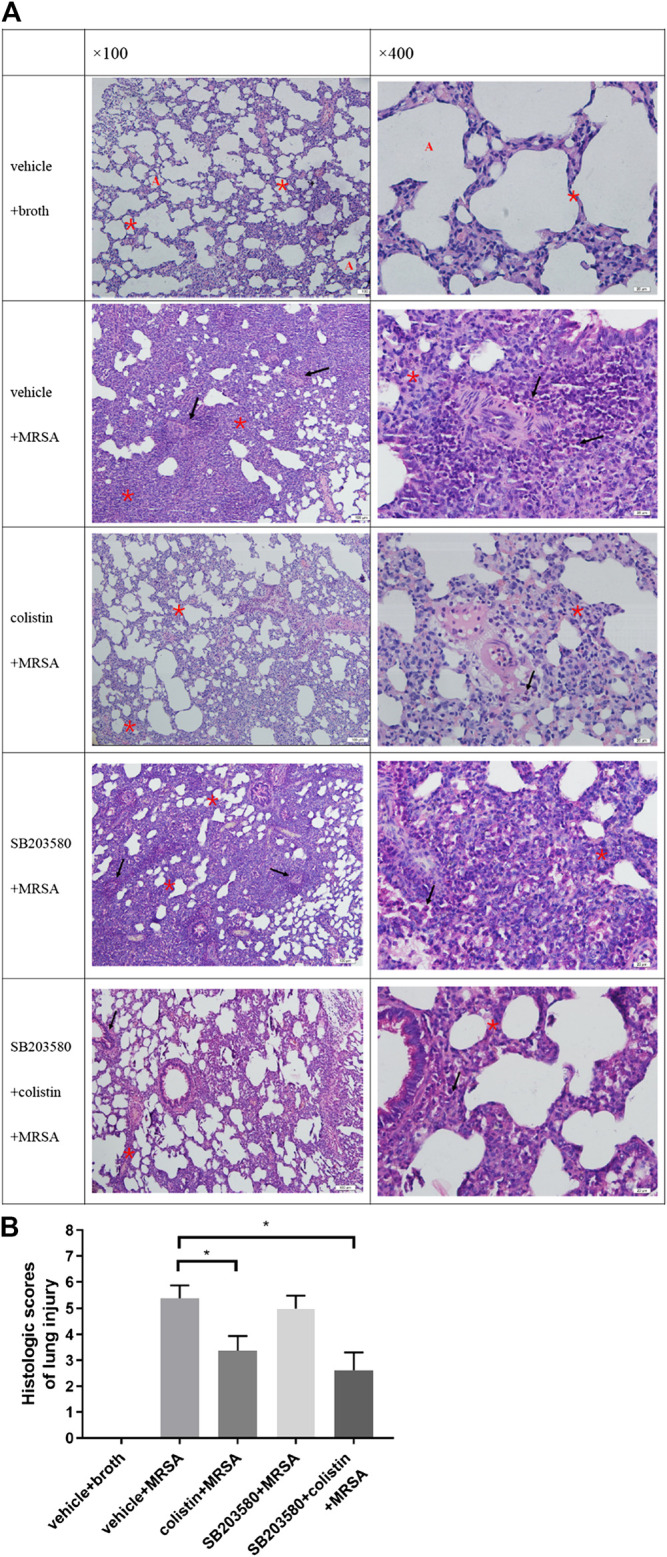
Histopathological changes in lung tissues. **(A)** HE staining at 100× and 400× magnification (100 μm, 20 μm). Normal alveolar structure (red A), infiltration of inflammatory cells (black arrow), alveolar septal thickening (red asterisks); **(B)** Lung injury score. Negative control group: vehicle + broth (n = 6); positive control group: vehicle + MRSA (n = 8); colistin group: colistin + MRSA (n = 8); SB203580 group: SB203580 + MRSA (n = 9); SB203580 + colistin group: SB203580 + colistin + MRSA (n = 8). Data are presented as mean ± SEM, *p < 0.05 compared to positive control group.

The lung histopathology of all rats that survived to 72 h after infection was evaluated (Figure 5A). In the negative control group (uninfected), lung tissues showed normal alveolar structure without inflammation. Compared with the uninfected rats, rats in all infected groups developed pneumonia with obvious internal alveolar septum thickening, while a significant cellular inflammatory response was observed, evidenced by diffuse inflammatory infiltrates with neutrophils. Disruption of alveoli was more frequently observed in the positive control and SB203580 groups, as well as a dominant polymorphic cell infiltration. Colistin and SB203580 + colistin groups trended to have less alveolar septum thickening and reduced cellular inflammatory infiltrates. Figure 5B demonstrates the lung injury score of each group. Rats in the negative control group had no inflammation with the injury score of 0. Positive control group had the highest injury score at 5.13, followed by the SB203580 group with an injury score of 5.00. Injury scores of the colistin and SB203580 + colistin groups were 3.75 and 2.63, respectively, both of which were significantly lower compared with the positive control group. There was no difference between the colistin and SB203580 + colistin groups.

**FIGURE 6:**
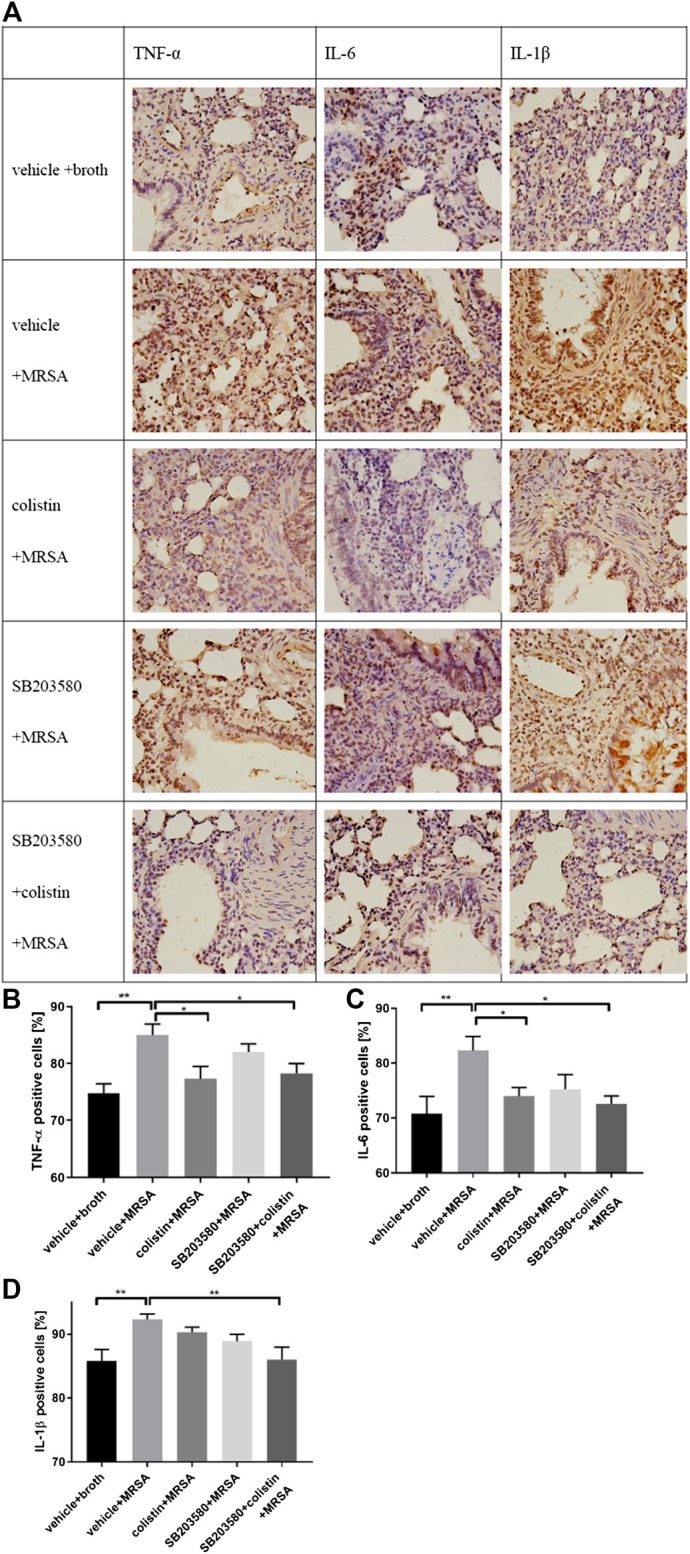
IHC staining to determine TNF-α, IL-6 and IL-1β expression in rat lung tissue. **(A)** Representative images of IHC staining for TNF-α, IL-6 and IL-1β; **(B)** Positive expression of TNF-α; **(C)** Positive expression of IL-6; **(D)** Positive expression of IL-1β. Data are presented as mean ± SEM, *p < 0.05 and **p < 0.01 compared to positive control group.

Cytokines, like TNF-α, IL-6 and IL-1β, in lung tissues were evaluated by IHC (Figure 6A). The proportions of TNF-α, IL-6 and IL-1β positive cells were significantly increased in the positive control group compared with the negative control group (Figures 6A,B,C). The levels of TNF-α and IL-6 in the colistin and SB203580 + colistin groups were obviously decreased compared with the positive control group, while no difference was observed between these two groups (Figures 6B,C). Moreover, the IL-1β level in the SB203580 + colistin group was lower compared with the positive control group, while no difference was observed in the colistin and SB203580 groups compared with the positive or negative control group (Figure 6D).

## Discussion

Using an experimental rat model of MRSA-induced pneumonia, we found that the administration of a single-dose colistin exerted immunomodulatory effects and ameliorated lung damage induced by MRSA infection in adult rats.

We started with the two regimens in order to explore the dose of colistin that can maximize the activation of immunoregulation. The inflammatory parameters did not change significantly in the twice-dosage group compared to single-dosage group. There might be two reasons for the twice-dosage group did not show immunoregulation effect. One is that when the same amount of colistin was divided into two dosage, concentrations of colistin in plasma or tissues did not reach the minimal effective concentration. The other reason is that 12 h after second dosage is not appropriate to observe the immunomodulatory effect. This is also consistent with our previous findings in *C. elegans* (Cai et al., 2014), which indicate a short, nontoxic treatment with colistin can activate the innate immunity of *C. elegans*. Therefore, we selected the single-dosage regimen of colistin and 6 h as the time point of sampling. In the present study, we chose three dosages of colistin, which are usually applied in rat infection model or colistin-induced nephrotoxicity model (Pantopoulou et al., 2007; Yousef et al., 2012; Ajiboye, 2018). The total WBC and granulocyte counts of colistin-treated healthy rats were increased in a dose-dependent manner. Moreover, the 9 mg/kg colistin dose could enhance the cytokine levels in plasma. As terminally differentiated phagocytes, neutrophils and granulocytes comprise more than half of all circulating WBCs, and act as first line of defense against invading microorganisms (Storisteanu et al., 2017). Once activated, neutrophils start pathogen killing process by elaborating an arsenal of antimicrobial peptides, reactive oxygen species (ROS) and proteases (Harrison, 2009). Our results indicated that colistin exerted its immunomodulatory effect on healthy rats by increasing the levels of granulocytes and cytokines. However, the duration of such effect was short, and it only lasted about 6 h after the first administration. At 12 h after the second dosage of colistin, the WBC counts and differential counts were similar compared with the control group. These results were consistent with our previous *in vitro* studies that 6 h colistin treatment significantly increases the secretion of TNF-α, IL-1β and IL-6 in macrophages from healthy rats (Wang et al., 2019).

While in MRSA-induced pneumonia rat model, the WBC and granulocyte counts were similar compared with the negative control group at 72 h after MRSA infection. This might be attributed to that rats were no longer in the early stages of infection. MRSA possesses an arsenal of virulence factors, many of which are employed to evade and counteract the host immune system (Thammavongsa et al., 2015). In the early stage of infection, activated innate immune cells initiate a significant increase in both innate immune and inflammatory responses to clear invading pathogens from the host (De la Calle et al., 2016). When the infection gets worse, especially when it develops into sepsis, even if patients survive the initial pro-inflammatory episode, they will experience subsequent challenge of immunosuppression. The immunosuppression is characterized by impaired innate and adaptive immune responses, including enhanced apoptosis and dysfunction of lymphocytes and impaired phagocyte functions (Muenzer et al., 2010). Apoptosis in microbial infections promotes the removal of the microorganisms, but often causes severe tissue damage in the meanwhile (Srinivasan et al., 2014). The characteristic cell changes during apoptosis, including blebbing, cell shrinkage, nuclear fragmentation, chromatin condensation, and chromosomal DNA fragmentation, could eventually lead to cell death and cell counts reduction (Lam et al., 2017). It was worth noting that the numbers of WBCs and granulocytes in the colistin group were still significantly higher than those in the positive control group. Moreover, pre-administration of only one dose of colistin displayed beneficial effects on infected lung tissues. Since MRSA is naturally resistant to colistin, the reason for the decline in colony counts was most likely attributed to the killing effect of activated immune cells on bacteria. Our results clearly showed that the number of immune cells increased, while the lung tissue injury and lung tissue burdens were reduced. We speculated that this was caused by the immunomodulatory effect of colistin, which could partially prevent the apoptosis of immune cells and protect the function of phagocytic cells.

The cytokines signaling network plays a vital role in modulation of inflammatory responses to eliminate pathogens. Proinflammatory cytokines (TNF-α, IL-6 and IL-1β) are considered as major inducers of the inflammatory response and involved in systemic response and local injury during pneumonia (Lee et al., 2010; Bordon et al., 2013). If the initial response is not properly controlled, it will result in exaggerated innate immune and inflammatory responses that can damage organs (Kakihana et al., 2016; Mira et al., 2017). In our study, the changes of cytokines in plasma were slightly different from those in lung tissues. In plasma, except that the IL-1β level in the positive control group was significantly higher compared with the negative control group, other cytokines remained at the same levels as the negative control group. In lung tissues, the TNF-α, IL-1β and IL-6 levels in the positive control group were all significantly higher compared with the negative control group at 72 h after infection. Kaku N. et al. (Kaku et al., 2016) have reported a significant increase in plasma cytokines (TNF-α, IL-1β and IL-6) in the short term (24 h) after MRSA infection. Jacqueline C. et al. (Jacqueline et al., 2014) have found that the TNF-α and IL-6 levels in mouse lung tissue homogenates are significantly increased in the early stages of MRSA infection, with a peak at 8 h after the bacterial challenge. After the rapid increase stage, each cytokine level was declined rapidly, although the levels of cytokines at 48 h were still higher compared with the negative control group. These results demonstrated the transient and kinetic aspects of signaling molecules involved in the immune response. In colistin-treated animals, the plasma levels of IL-1β and IL-6 were decreased compared with the positive control group. Moreover, the MRSA-induced up-regulation of TNF-α and IL-6 was also inhibited with the colistin treatment in lung tissues from IHC staining. Our results were consistent with published data that colistin can reduce the lipopolysaccharide (LPS)-triggered up-regulation of inflammatory cytokines. Nanjo Y. (Nanjo et al., 2013) has suggested that the use of colistin sulfate (subcutaneously injected at 1.0 mg per mouse) can reduce the levels of TNF-α, IL-6 and IL-1β in LPS-induced sepsis mouse model and lead to decreased toxicity. Matzneller P. (Matzneller et al., 2017) has performed a randomized, double-blinded, placebo-controlled crossover trial to determine the degree of colistin-driven inflammatory response in blood of LPS-challenged healthy volunteers. After a single intravenous dose of 2.5 million IU colistin methane sulfonate, the levels of IL-6, IL-8 and TNF-α have significantly decreased in blood of healthy volunteers. This effect is most evident for IL-6, IL-8, and TNF-α. Increasing evidence confirms that cytokine balance is crucial to outcomes for patients with pneumonia (Park et al., 2001; de Brito et al., 2016). In this case, if the balance cannot be maintained within a certain range, deleterious effects can increase the severity of pneumonia. Our results indicated that colistin had a protective effect on the development of pulmonary inflammation by delaying the continuous up-regulation of pro-inflammatory factors, which was important in the complex cytokine balance.

Histopathological results clearly showed that colistin possessed significant superiority over the groups without colistin. There might be two reasons for this result. First, early activation of immune cells increased the clearance of pathogens from lung tissue. Secondly, colistin prevented the excessive up-regulation of cytokines during late infection, which was an important factor for lung tissue injuries.

In our previous study, we found that colistin is capable of activating the innate immunity in a conserved p38/PMK-1 dependent manner when using *C. elegans* as a model organism. The protection against bacterial infections induced by colistin can be completely abolished by PMK-1 inhibition (Cai et al., 2014). Next, it is found in the study of mammalian macrophages that colistin significantly increases the secretion of pro-inflammatory cytokines and phagocytotic ability. The increased phagocytosis can be blocked by p38 inhibitor SB203580 (Wang et al., 2019). Microarray analyses have revealed that except for mitogen-activated protein kinase (MAPK), mammalian target of rapamycin (mTOR), chemokine and B cell receptor pathways are also greatly impacted during colistin stimulation process (Wang et al., 2019). However, we did not find that SB203580 could fully block the reduction of cytokines in plasma and lung tissues and aggravation of lung injuries caused by colistin. This result was not unexpected, because the immune system of mammals is more complex than that of *C. elegans*. Previous research has also suggested that multiple pathways participate in the immune regulatory response caused by colistin. Therefore, it is unlikely to achieve a significant blocking effect by inhibiting only one of these pathways.

There are limitations in the present study. First, time-dependent dynamic changes of cytokines or lung injuries in infected animals could not be described based on the data obtained so far. Secondly, although the pro-inflammatory cytokines were controlled by colistin regimen, it remains unknown whether the anti-inflammatory cytokines are affected at the same time.

## Conclusion

In conclusion, the data presented here suggested that colistin, which was confirmed to have immunomodulatory effect, could attenuate an excessive inflammatory reaction and protect the lung from MRSA-associated injuries. Moreover, p38/MAPK was not the only pathway involved in these effects. The in-depth mechanism underlying the immunomodulatory effect of colistin in mammals needs to be further explored.

## Data Availability Statement

The original contributions presented in the study are included in the article/Supplementary Material, further inquiries can be directed to the corresponding author.

## Ethics Statement

The animal study was reviewed and approved by Animal Care and Use Committee (IACUC) of PLA General Hospital.

## Author Contributions

HN and TY contributed to the laboratory data acquisition, data analysis and drafting of article. JW and RW contributed to the design of study and critical revision. YC contributed to the conception and design of study, analysis of data and drafting of article and critical revision.

## Funding

This work was supported by the National Natural Science Foundation of China (Nos. 81573472 and 81770004) and 13th Five-Year Plan of National Major Science and Technology Projects of China (No. 2018ZX09201-013).

## Conflict of Interest

The authors declare that the research was conducted in the absence of any commercial or financial relationships that could be construed as a potential conflict of interest.
